# Targeting Hypoxia-A2A Adenosinergic Immunosuppression of Antitumor T Cells During Cancer Immunotherapy

**DOI:** 10.3389/fimmu.2020.570041

**Published:** 2020-09-29

**Authors:** Joseph M. Steingold, Stephen M. Hatfield

**Affiliations:** Department of Pharmaceutical Sciences, New England Inflammation and Tissue Protection Institute, Bouvé College of Health Sciences, Northeastern University, Boston, MA, United States

**Keywords:** adenosine, hypoxia, cancer immunotherapies, T cell, HIF−1α, immune checkpoint, immunology

## Abstract

The blockade of immunological negative regulators offered a novel therapeutic approach that revolutionized the immunotherapy of cancer. Still, a significant portion of patients fail to respond to anti-PD-1/PD-L1 and/or anti-CTLA-4 therapy or experience significant adverse effects. We propose that one of the major reasons that many patients do not respond to this form of therapy is due to the powerful physiological suppression mediated by hypoxia-adenosinergic signaling. Indeed, both inflamed and cancerous tissues are hypoxic and rich in extracellular adenosine, in part due to stabilization of the transcription factor hypoxia-inducible factor 1 alpha (HIF-1α). Adenosine signals through adenosine A2A receptors (A2AR) to suppress anti-tumor and anti-pathogen immune responses. Several classes of anti-hypoxia-A2AR therapeutics have been offered to refractory cancer patients, with A2AR blockers, inhibitors of adenosine-generating enzymes such as CD39 and CD73, and hypoxia-targeting drugs now reaching the clinical stage. Clinical results have confirmed preclinical observations that blockade of the hypoxia-adenosine-A2AR axis synergizes with inhibitors of immune checkpoints to induce tumor rejection. Thus, A2AR blockers provide a new hope for the majority of patients who are nonresponsive to current immunotherapeutic approaches including checkpoint blockade. Here, we discuss the discoveries that firmly implicate the A2AR as a critical and non-redundant biochemical negative regulator of the immune response and highlight the importance of targeting the hypoxia-adenosine-A2AR axis to manipulate anti-pathogen and anti-tumor immune responses.

## Overview of the Hypoxia-Adenosine-A2AR Axis

While hypoxia-dependent generation of extracellular adenosine and subsequent immunosuppressive signaling through adenosine A2A receptors (A2AR) is deleterious in the tumor microenvironment (TME), this mechanism normally has an important tissue-protective function. The suppression of tumor-reactive T cells by hypoxia-adenosine-A2AR signaling in the TME is a commandeering of this evolutionarily conserved, non-redundant feedback mechanism to govern inflammation (1–3). Sitkovsky and colleagues were the first to confirm *in vivo* that this may explain the paradoxical peaceful coexistence of tumors and antitumor T cells in tumors (4, 5). These studies demonstrated that A2AR signaling inhibited important effector functions of T cells, such as secretion of pro-inflammatory cytokines (e.g., IFNγ) (6). However, the anti-inflammatory effects of the hypoxia-adenosine-A2AR axis have been confirmed and extended to include suppression of T cell proliferation, cytotoxicity, and induction of anti-inflammatory cytokine secretion (e.g., IL-10) (7–9).

The hypoxia-adenosine-A2AR axis of immunosuppression begins with hypoxia and the stabilization of hypoxia-inducible factor-1alpha (HIF-1α), which increases extracellular adenosine in part by upregulating adenosine-generating enzymes. Subsequent signaling through the Gs-coupled/cAMP-elevating A2ARs induces protein kinase A (PKA)-mediated inhibition of T-cell receptor signaling and immunosuppressive transcriptional changes (10). This includes the inhibition of pro-inflammatory cytokine secretion and an increase in the levels of anti-inflammatory cytokines that contain a cAMP response element (CRE) consensus sequence in their respective promoter regions. While adenosine can also activate cAMP-elevating adenosine A2B receptors (A2BRs), our research has focused on A2AR adenosinergic immunosuppression due to a higher affinity for adenosine and higher expression on T cells (11–13). Importantly, A2AR expression seems to be the limiting factor in adenosine-mediated cAMP generation in T cells since there is no receptor reserve of A2AR (14). T cells can also possess a memory of A2AR signaling, allowing the effects of adenosine to persist long after exposure (15).

Adenosine also exerts immunosuppressive effects through A2BR, particularly on innate immune cells. Groundwork for this hypothesis can be found in studies demonstrating that adenosinergic immunosuppression of IL-12 and TNFα by macrophages is at least partially A2AR-independent (16). For example, in lipopolysaccharide-stimulated macrophages, A2BR activation increases anti-inflammatory IL-10 production by attenuating translational arrest of IL-10 mRNA (17). Conversely, A2BR signaling may enhance activation of alternative/Th2 cytokine-activated macrophages, which manifest several anti-inflammatory functions (18). In group 2 innate lymphoid cells (IL2C), adenosine has been demonstrated to decrease IL-5 and IL-13 production through A2BR, but increase IL-5 production through A2AR. Activation of both A2AR and A2BR in IL2C results in a net decrease in IL-5 production, indicating the importance of A2BR on this cell type (19). Interestingly, HIF-1α-dependent expression of A2BR has also been shown to induce the enrichment of breast cancer stem cells (20). Additional studies of preclinical models of acute lung injury have also demonstrated that an increase in HIF-1α levels in pulmonary epithelia subjected to cyclic mechanical stretch resulted in an increase in A2BR expression (21). A2BR-mediated immunosuppression of a variety of immune cells, including dendritic cells, has led to the development of dual A2AR/A2BR antagonists which may prevent adenosinergic immunosuppression of both innate and adaptive immune cells (22).

The main metabolic precursor to adenosine is ATP. Under homeostatic conditions, ATP is magnitudes higher intracellularly than in the extracellular space (23, 24). However, in inflamed and cancerous tissues, apoptotic and necrotic cells release ATP into the extracellular compartment, disrupting this gradient (25). Excess ATP is then degraded into adenosine by CD39/CD73 (26–29), CD38/CD203a (30–33) and other phosphatases in certain tissues (28). While the primary mechanism is thought to be mediated by CD39 and CD73 (34), alternative adenosine-generating pathways, such as CD38, are an important contributor to adenosine levels in the TME and inhibit antitumor T cells via A2AR. Indeed, recent studies have demonstrated that PD-1 blockade can increase CD38 expression, leading to resistance to αPD-1 therapy (35).

Consistent with findings regarding adenosine-A2AR immunosuppression, multiple studies from different teams have confirmed the tissue-protecting roles of CD39 and CD73. CD39, which converts ATP to AMP, also serves an anticoagulant function in vasculature (36). Indeed, CD39 has been demonstrated to attenuate both renal ischemia and acute lung injury (37, 38). CD73, which converts AMP to adenosine, has also been shown to have a role in the mediation of cell adhesion to endothelium (39). Moreover, some tumorigenic functions of CD73 have been shown to be independent of its enzymatic function, such as induction of angiogenesis (40). Interestingly, recent studies have also shown that A2AR signaling can promote angiogenesis, suggesting a role for the HIF-1α-CD73-adenosine-A2AR axis in tumor-associated lymphangiogenesis and metastasis (41).

The upstream portion of the hypoxia-adenosine-A2AR axis is mediated by hypoxia/HIF-1α. HIF-1α upregulates genes containing an hypoxia response element (HRE) consensus sequence that mediates cell survival in hypoxic conditions. The immunosuppressive role of HIF-1α was first implicated in studies of HIF-1α^−/−^ Rag-2^−/−^ mice with HIF-1α deletion in T cells and B cells. These experiments demonstrated that HIF-1α regulates lymphocyte development and prevents autoimmunity (42). Subsequent studies of mice with T cell-specific HIF-1α deletion confirmed an immunosuppressive role for HIF-1α. These mice exhibited an enhanced antibacterial response due to the lack of HIF-1α-mediated inhibition of T cells (43). Studies that prevent HIF-1α stabilization using supplemental oxygenation have also provided direct mechanistic evidence for HIF-1α-mediated upregulation of the hypoxia-adenosine-A2AR axis (44). It must be emphasized that upregulation of CRE-containing genes and HRE-containing genes may not be mutually exclusive. The gene encoding the characteristic regulatory T-cell transcription factor FoxP3, which upregulates HIF-1α, is induced by CRE activation (45, 46). Thus, it is suggested that crosstalk exists between CRE and HRE pathways and they may synergize to strengthen immunosuppression (47, 48). Physiologically, this is supported by the infectious tolerance mediated by regulatory T cells in inflamed and cancerous tissues (49–51).

## Pharmacological Targets in the Hypoxia-Adenosine-A2AR Axis for Cancer Immunotherapy

### A2ARs

Inquiry into the immunosuppressive functions of adenosine was catalyzed by the established importance of cAMP as an immunosuppressive agent (52). cAMP has been demonstrated to inhibit many effector T cell functions via PKA activation (53–59). Landmark studies by Sitkovsky provided the first genetic and pharmacological evidence that the cAMP-elevating A2AR has a critical and non-redundant immunosuppressive role in tissue protection during excessive inflammation (6). These studies also offered insights into why antitumor T cells often fail to mount an effective response against cancerous tissue. Indeed, tumors are rich in extracellular adenosine, in large part due to poor, irregular vasculature resulting in local hypoxia (60–62). The tumor-protecting role of A2AR was conclusively established using mice with A2AR gene deletion (5). This study also complemented genetic evidence with pharmacological data, demonstrating that A2AR antagonism or silencing by siRNA enhanced the efficacy of adoptive cell transfer (ACT) (5). This was supported by follow-up studies demonstrating that A2AR antagonism during ACT or adoptive transfer of A2AR-deficient T cells were effective approaches for enhancing the efficacy of ACT in mice (63). The therapeutic benefit of A2AR antagonism was shown to be due in part by increased IFNγ secretion by tumor-infiltrating adoptively transferred T cells (63). Importantly, this study also demonstrated that A2AR antagonism improved anti-tumor immunity independent of the anatomical location of the tumor and provided long-term tumor-specific memory (63). Taken together, these studies provided proof of principal for the use of A2AR antagonists during cancer immunotherapies, particularly ACT.

The progress in methods of ACT and the studies reviewed above offered justification to test whether CAR-T cells might also be susceptible to hypoxia-adenosinergic immunosuppression. It has been hypothesized that A2AR blockade may improve efficacy of CAR-T therapies against cancers. This may prove essential for CAR-T that target solid tumors, which are known to be hypoxic and extracellular adenosine-rich. Indeed, early evidence was provided by Albelda's group demonstrating that genetic engineering to prevent PKA trafficking to the CAR-T cell membrane enhanced antitumor function *in vivo* and conferred resistance to adenosinergic immunosuppression *in vitro* (64). Critical studies by Darcy's Team demonstrated that both pharmacological and genetic inhibition of A2AR enhanced CAR-T efficacy in two distinct murine models of syngeneic breast cancer. Of clinical relevance, addition of αPD-1 to the CAR-T/A2AR blockade protocol further enhanced CAR-T efficacy, as indicated by increased IFNγ production by CAR-T (65). These findings confirm and extend the observations that A2AR antagonism enhances production of IFNγ by polyclonal adoptively transferred T cells in the TME to improve tumor regression (63).

Pioneering studies by Powell's Team established that A2AR agonism can upregulate negative regulators of the immune response such as LAG-3 (8). Subsequent studies using the A2AR antagonist CPI-444 have also provided strong justification for A2AR blockade during cancer immunotherapies. These studies confirmed and extended observations of improved antitumor efficacy of ACT in combination with A2AR blockade. Additional mechanistic evidence justifying A2AR blockade was provided by demonstrations that A2AR blockade reduced PD-1 and LAG-3 expression on effector and regulatory T cells, as well as reduced expression of these immune checkpoint molecules in tumor-draining lymph nodes (66). Taken together, these findings indicate that A2AR blockade can prevent inhibition of already active antitumor T cells, and also prevent inhibition during initial activation (66). Consistent with this finding, it has also been demonstrated that A2AR deletion increases terminally mature natural killer cells in the TME, implicating adenosine as a negative regulator of innate immune cell maturation as well (67). Important studies by Miller and Willingham in multiple preclinical cancer models confirmed that combining A2AR antagonism with checkpoint blockade improved tumor regression, strengthening mechanistic evidence to justify clinical testing of this approach (68). *In vitro* assays also demonstrated that CPI-444 prevented adenosinergic inhibition of IL-2 and IFNγ production by T cells (68). Through analysis of gene expression, these studies were also able to identify a Th1 expression signature that was associated with positive responses to dual blockade of A2AR/PD-L1 (68).

These preclinical studies have led to the clinical testing of A2AR antagonists as a cancer therapy and have yielded promising results. Against renal cell cancer, A2AR antagonism using CPI-444 induced durable responses both as a monotherapy and when combined with the PD-L1 inhibitor atezolizumab. Patients experiencing positive responses included individuals who had previously shown resistance to αPD-L1 therapy. Consistent with preclinical data, alleviation of adenosinergic immunosuppression resulted in higher cytotoxic T cell tumor infiltration. This study also elucidated a gene-expression signature that was associated with positive response (69). In another clinical study, the A2AR antagonist NIR178 administered both as a monotherapy and in combination with the PD-1 inhibitor spartalizumab to 24 non-small lung cancer patients resulted in stable disease in fifteen patients in addition to one partial response and one complete response (70). Furthermore, the A2AR antagonist AZD4635 used as a monotherapy and in combination with the PD-L1 inhibitor durvalumab induced strong responses in three of eight metastatic castration-resistant prostate cancer patients (71). These tumors may be naturally adenosine-rich due to prostatic acid phosphatase activity and therefore a good candidate for A2AR blockade (71).

### CD39/CD73

It has been established that CD39/CD73 also have a major role in facilitating immune escape by tumors. Indeed, Robson's Team established the field of CD39 and were the first to demonstrate that CD39 deletion alleviated tumor burden in a preclinical model of hepatic metastatic cancer (72). Parallel studies by Smyth's Team also demonstrated that administration of a CD73 monoclonal antibody (mAb) decreased tumor burden in two distinct murine tumor models. This approach also suggested that not only did CD73 inhibit antitumor leukocytes via adenosine generation, but affected tumor metastasis as well (73). Moreover, Stagg's Team demonstrated that CD73 overexpression in human triple-negative breast cancer correlated with poor prognosis and resistance to chemotherapy in a preclinical model of breast cancer (74). Important studies by Smyth's Team also demonstrated improved anti-tumor efficacy using an A2AR antagonist in combination with a CD73 inhibitor to alleviate tumor burden (75). These findings also highlight the importance of targeting multiple components of the hypoxia-adenosine-A2AR axis. Indeed, small molecule inhibitors or monoclonal antibodies against CD39 and CD73 are emerging as potent anti-cancer therapies (49, 74, 76–82). Furthermore, αCD73 therapy has been demonstrated to improve the therapeutic benefit of αPD-1/αCTLA-4 therapy in multiple preclinical cancer models (80).

Several mAb CD73 inhibitors have exhibited strong antitumor efficacy in clinical trials with findings consistent with preclinical data. In 66 pancreatic or colorectal cancer patients, the αCD73 mAb MEDI9447 as monotherapy and in combination with durvalumab decreased CD73 expression on peripheral T cells. In addition, MEDI9447 decreased CD73 expression in five out of nine tumors, which correlated with increased cytotoxic T cell infiltration (83). The αCD73 mAb BMS986179 as a monotherapy and in combination with the PD-1 inhibitor nivolumab also induced partial responses or stable disease in 17 of 59 patients with various malignancies (84).

### HIF-1α

Given the hypoxia-HIF-1α-mediated upregulation of adenosine-generating enzymes, Sitkovsky's Team established in decades-long studies that hypoxia-HIF-1α inhibits T cells (10). It was then hypothesized and confirmed that the reversal of hypoxia could prevent the inhibition of antitumor T cells by hypoxia-adenosine-A2AR-mediated immunosuppression. Indeed, preclinical studies demonstrated that supplemental oxygen (60% O_2_) decreased levels of hypoxia, HIF-1α, and extracellular adenosine in the TME (44). This was supported by data demonstrating oxygenation-mediated reduction in CD39, CD73, A2AR, A2BR, and COX-2 expression (44). Importantly, supplemental oxygen was also shown to upregulate MHC class I expression by tumor cells, allowing for increased recognition and subsequent elimination by antitumor T cells (44). Parallel studies demonstrated the immunological effects of supplemental oxygen by showing that oxygenation converts an immunosuppressive TME to an immunopermissive TME. This resulted in an increase in many pro-inflammatory cytokines as well as recruitment of endogenous and adoptively transferred antitumor T cells into the TME. This was also accompanied by a reduction in many anti-inflammatory molecules such as TGFβ, CTLA-4, and FoxP3, as well as an overall reduction in regulatory T cells in the TME (85). This resulted in significant tumor regression and long-term survival in preclinical tumor models. Importantly, these studies also established that the reversal of hypoxia improved the efficacy of immune checkpoint blockade with αCTLA-4/αPD-1 (85).

HIF-1α can also be pharmacologically targeted using small molecule drugs such as digoxin, acriflavine, and ganetespib. Indeed, these drugs have shown efficacy in preclinical tumor models (86–88). While the immunosuppressive effects of HIF-1α have been shown to be mediated in part by hypoxia-adenosinergic signaling, HIF-1α also has other non-adenosinergic immunosuppressive effects (89). Additionally, immunosuppression via adenosine-A2AR signaling may not be completely reversed by only targeting hypoxia/HIF-1α. Therefore, an ideal approach for completely abrogating the immunosuppressive effects of the hypoxia-adenosine-A2AR axis might be the co-administration of both anti-hypoxia-HIF-1α therapies and A2AR antagonists during cancer immunotherapy (90).

## Conclusion

The hypoxia-adenosine-A2AR axis is a potent inhibitor of antitumor T cells. This pathway presents multiple pharmacological targets. Of particular importance and translational value are A2ARs, CD39/CD73, and HIF-1α. Inhibition of this pathway has been shown to enhance the efficacy of current cancer immunotherapy approaches, including αCTLA-4/αPD-1. Multiple studies have reported synergism between checkpoint inhibitors and several classes of anti-hypoxia-adenosine-A2AR therapeutics. Our preclinical studies provided the rationale and justification for combining A2AR blockade and supplemental oxygen/oxygenation agents during cancer immunotherapies. We postulate that this approach will maximize the efficacy of the antitumor immune response in clinical studies.

## Author Contributions

All authors listed have made a substantial, direct and intellectual contribution to the work, and approved it for publication.

## Conflict of Interest

The authors declare that the research was conducted in the absence of any commercial or financial relationships that could be construed as a potential conflict of interest.
